# Links between Fibrogenesis and Cancer: Mechanistic and Therapeutic Challenges

**DOI:** 10.3390/ijms20174313

**Published:** 2019-09-03

**Authors:** Liqiang Qin, Esteban C. Gabazza

**Affiliations:** 1 Department of Nephrology, Wenzhou Medical University, Wenzhou 317000, China; 2 Department of Immunology, Mie University School of Medicine, Edobashi 2-174, Tsu-city, Mie 514-8507, Japan

Fibrosis is the end-stage of chronic inflammatory diseases and tissue damage resulting from a dysregulated wound-healing response [1]. Most organs can develop scarring tissue in association with different pathological states including chronic inflammation, autoimmune disorders, graft rejection, malignant tumors or unknown factors [1]. Organ fibrosis is the cause of about 45% of the worldwide total death in developed countries [2]. The high frequency of co-morbidities, the increased mortality rate, the enigma surrounding the disease pathogenesis and the lack of effective therapeutic modalities have made organ fibrosis an area of great interest and consequently the focus of many investigations in recent years [3,4]. This special issue, “Links between Fibrogenesis and Cancer: Mechanistic and Therapeutic Challenges”, depicts original findings on intracellular signaling pathways playing a role in fibrogenesis and carcinogenesis associated with chronic diseases and metabolic disorders, and includes reviews describing recent discoveries on potential pathogenic factors, pathophysiological insights, and novel treatment options.

The propensity of fibrotic tissue to develop cancer is an old story, although the mechanistic pathways are still not completely understood [5]. The pro-tumorigenic microenvironment is characterized by the occurrence of persistent parenchymal cell death, compensatory regenerative overresponse, genome instability, DNA damage and gene mutations, and by an excessive local release of inflammatory cytokines, growth factors, and reactive oxygen species [5]. Liver cirrhosis caused by non-alcoholic steatohepatitis or chronic hepatitis B and C infections is an example of scarring disorder that ends up developing cancer [6,7,8]. As reviewed in this collection, new advances in the technology of next-generation sequencing and whole-genome sequencing have provided a huge amount of information on genetic aberrations during non-alcoholic steatohepatitis or chronic hepatitis B and C infections that trigger the activation of several intracellular oncogenic pathways [7,8]. Of these, disruption of the Hippo and the Yes-associated protein/transcriptional coactivator with PDZ-binding motif signaling pathways appears to be a critical step in the process of hepatic fibrogenesis, hepatic carcinogenesis, and resistance to therapy, angiogenesis, and proliferation of cancer-associated fibroblasts [4,9]. In established malignant tumors, the surrounding tumor microenvironment is a rich source of factors causing uncontrolled activation of intracellular pathways in cancer cells [10]. Components of the tumor microenvironment such as cancer-associated fibroblasts, macrophages, lymphocytes, endothelial cells, and the extracellular matrix may weaken the host immune response and promote the invasiveness and metastatic potential of hepatocellular carcinoma cells directly by interacting with cancer cells or indirectly by releasing an excessive amount of regulatory and suppressive factors [11,12]. The tumor microenvironment may also induce resistance to anti-cancer therapy and, as a proof-of-concept, recent studies have shown amelioration of therapeutic response to anti-cancer drugs by treatment with tumor microenvironment-modifying agents [12].

Another morbid and frequent association described here is idiopathic pulmonary fibrosis (IPF) and lung cancer [13]. Idiopathic pulmonary fibrosis is a chronic and devastating disease of unknown etiology [14]. There are similarities between IPF and cancer in terms of clinical behavior and molecular pathways underlying the pathogenesis of the disease [13,15,16]. Both diseases are characterized by poor response to therapy, short life expectancy, and frequent exposure to harmful environmental factors (e.g., cigarette smoke) [16]. Indeed, the prognosis of idiopathic pulmonary fibrosis is poorer than most types of cancer except malignant tumors of the lung or pancreas [17]. As outlined in this special issue, idiopathic pulmonary fibrosis shares common features with cancer in terms of genetic and epigenetic changes, abnormalities in cell-to-cell communication, aberrant activation of intracellular signal pathways, deregulated expression of growth factors/proteolytic enzymes, uncontrolled cell apoptosis and activation of pro-fibrotic cells [13]. This concept of close-identity between aberrant tissue scarring and cancer is not restricted to the lungs. Although at variable levels, most organs with either fibrotic, benign or malignant disease show abnormal activation of similar signaling and mechanistic pathways [15,18,19,20]. Fibroblast is one of these common components of cancer and fibrotic disease that plays detrimental roles by contributing to disease progression [6,10,11,12,21]. Cancer- and scarring tissue-associated fibroblasts may further differentiate to myofibroblasts by cytokines and growth factors such as transforming growth factorβ1 to expand the desmoplastic tissue in both pathologies and to promote cell invasiveness and resistance to therapy in malignant tumors [6,10,11]. Studies evaluating the phenotypic and behavioral alterations of cellular mediators, and biochemical and molecular abnormalities common to organ injury, fibrosis and malignant diseases may provide hints for the development of new diagnostic approaches and effective and less toxic therapeutic modalities for both diseased conditions [22,23,24,25,26].

Recent discoveries in science and advances in technology have revolutionized the diagnostic procedures and therapeutic approaches of many diseases leading to a significant amelioration of quality-of-life and prognosis. However, the incidence and burden of organ fibrosis and cancer are still increasing worldwide [27,28,29]. Much work remains to be done to achieve more timely diagnosis and to improve the therapeutic management of fibrotic and malignant diseases. The recent insights into the pathogenesis of organ fibrogenesis and malignancy and the new potential therapeutic targets described in the present special issue may be useful for identifying new biological markers and for drug discovery.

## References

[1] Wynn T.A. (2008). Cellular and molecular mechanisms of fibrosis. J. Pathol..

[2] Wynn T.A., Ramalingam T.R. (2012). Mechanisms of fibrosis: Therapeutic translation for fibrotic disease. Nat. Med..

[3] Cano-Jimenez E., Hernandez Gonzalez F., Peloche G.B. (2018). Comorbidities and Complications in Idiopathic Pulmonary Fibrosis. Med. Sci..

[4] Moon H., Cho K., Shin S., Kim D.Y., Han K.H., Ro S.W. (2019). High Risk of Hepatocellular Carcinoma Development in Fibrotic Liver: Role of the Hippo-YAP/TAZ Signaling Pathway. Int. J. Mol. Sci..

[5] Ballester B., Milara J., Cortijo J. (2019). Idiopathic Pulmonary Fibrosis and Lung Cancer: Mechanisms and Molecular Targets. Int. J. Mol. Sci..

[6] Baglieri J., Brenner D.A., Kisseleva T. (2019). The Role of Fibrosis and Liver-Associated Fibroblasts in the Pathogenesis of Hepatocellular Carcinoma. Int. J. Mol. Sci..

[7] Kanda T., Goto T., Hirotsu Y., Moriyama M., Omata M. (2019). Molecular Mechanisms Driving Progression of Liver Cirrhosis towards Hepatocellular Carcinoma in Chronic Hepatitis B and C Infections: A Review. Int. J. Mol. Sci..

[8] Sircana A., Paschetta E., Saba F., Molinaro F., Musso G. (2019). Recent Insight into the Role of Fibrosis in Nonalcoholic Steatohepatitis-Related Hepatocellular Carcinoma. Int. J. Mol. Sci..

[9] Noguchi S., Saito A., Nagase T. (2018). YAP/TAZ Signaling as a Molecular Link between Fibrosis and Cancer. Int. J. Mol. Sci..

[10] Hao Y., Baker D., Ten Dijke P. (2019). TGF-beta-Mediated Epithelial-Mesenchymal Transition and Cancer Metastasis. Int. J. Mol. Sci..

[11] Truffi M., Mazzucchelli S., Bonizzi A., Sorrentino L., Allevi R., Vanna R., Morasso C., Corsi F. (2019). Nano-Strategies to Target Breast Cancer-Associated Fibroblasts: Rearranging the Tumor Microenvironment to Achieve Antitumor Efficacy. Int. J. Mol. Sci..

[12] Yoshida G.J., Azuma A., Miura Y., Orimo A. (2019). Activated Fibroblast Program Orchestrates Tumor Initiation and Progression; Molecular Mechanisms and the Associated Therapeutic Strategies. Int. J. Mol. Sci..

[13] Kinoshita T., Goto T. (2019). Molecular Mechanisms of Pulmonary Fibrogenesis and Its Progression to Lung Cancer: A Review. Int. J. Mol. Sci..

[14] Barratt S.L., Creamer A., Hayton C., Chaudhuri N. (2018). Idiopathic Pulmonary Fibrosis (IPF): An Overview. J. Clin. Med..

[15] Tzouvelekis A., Gomatou G., Bouros E., Trigidou R., Tzilas V., Bouros D. (2019). Common Pathogenic Mechanisms Between Idiopathic Pulmonary Fibrosis and Lung Cancer. Chest.

[16] Vancheri C., Failla M., Crimi N., Raghu G. (2010). Idiopathic pulmonary fibrosis: A disease with similarities and links to cancer biology. Eur. Respir. J..

[17] du Bois R.M. (2012). An earlier and more confident diagnosis of idiopathic pulmonary fibrosis. Eur. Respir. Rev..

[18] Barcena-Varela M., Colyn L., Fernandez-Barrena M.G. (2019). Epigenetic Mechanisms in Hepatic Stellate Cell Activation During Liver Fibrosis and Carcinogenesis. Int. J. Mol. Sci..

[19] Ciebiera M., Wlodarczyk M., Zgliczynska M., Lukaszuk K., Meczekalski B., Kobierzycki C., Lozinski T., Jakiel G. (2018). The Role of Tumor Necrosis Factor alpha in the Biology of Uterine Fibroids and the Related Symptoms. Int. J. Mol. Sci..

[20] Islam M.S., Ciavattini A., Petraglia F., Castellucci M., Ciarmela P. (2018). Extracellular matrix in uterine leiomyoma pathogenesis: A potential target for future therapeutics. Hum. Reprod. Update.

[21] Liu T., Zhou L., Li D., Andl T., Zhang Y. (2019). Cancer-Associated Fibroblasts Build and Secure the Tumor Microenvironment. Front. Cell Dev. Biol..

[22] Baffour Tonto P., Yasuma T., Kobayashi T., D’Alessandro-Gabazza C.N., Toda M., Saiki H., Fujimoto H., Asayama K., Fujiwara K., Nishihama K. (2019). Protein S is Protective in Acute Lung Injury by Inhibiting Cell Apoptosis. Int. J. Mol. Sci..

[23] Chuang H.M., Ho L.I., Huang M.H., Huang K.L., Chiou T.W., Lin S.Z., Su H.L., Harn H.J. (2018). Non-Canonical Regulation of Type I Collagen through Promoter Binding of SOX2 and Its Contribution to Ameliorating Pulmonary Fibrosis by Butylidenephthalide. Int. J. Mol. Sci..

[24] On S., Kim H.Y., Kim H.S., Park J., Kang K.W. (2019). Involvement of G-Protein-Coupled Receptor 40 in the Inhibitory Effects of Docosahexaenoic Acid on SREBP1-Mediated Lipogenic Enzyme Expression in Primary Hepatocytes. Int. J. Mol. Sci..

[25] Rubisz P., Ciebiera M., Hirnle L., Zgliczynska M., Lozinski T., Dziegiel P., Kobierzycki C. (2019). The Usefulness of Immunohistochemistry in the Differential Diagnosis of Lesions Originating from the Myometrium. Int. J. Mol. Sci..

[26] Yasuma T., Kobayashi T., D’Alessandro-Gabazza C.N., Fujimoto H., Ito K., Nishii Y., Nishihama K., Baffour Tonto P., Takeshita A., Toda M. (2018). Renal Injury during Long-Term Crizotinib Therapy. Int. J. Mol. Sci..

[27] Hutchinson J., Fogarty A., Hubbard R., McKeever T. (2015). Global incidence and mortality of idiopathic pulmonary fibrosis: A systematic review. Eur. Respir. J..

[28] Marengo A., Rosso C., Bugianesi E. (2016). Liver Cancer: Connections with Obesity, Fatty Liver, and Cirrhosis. Annu. Rev. Med..

[29] Webster A.C., Nagler E.V., Morton R.L., Masson P. (2017). Chronic Kidney Disease. Lancet.

